# Gross Cystic Disease Fluid Protein-15/Prolactin-Inducible Protein as a Biomarker for Keratoconus Disease

**DOI:** 10.1371/journal.pone.0113310

**Published:** 2014-11-18

**Authors:** Shrestha Priyadarsini, Jesper Hjortdal, Akhee Sarker-Nag, Henrik Sejersen, John M. Asara, Dimitrios Karamichos

**Affiliations:** 1 Ophthalmology, University of Oklahoma - Dean McGee Eye Institute, Oklahoma City, Oklahoma, United States of America; 2 Department of Ophthalmology, Aarhus University Hospital, Aarhus C, Denmark; 3 Division of Signal Transduction/Mass Spectrometry Core, Beth Israel Deaconess Medical Center, Harvard Medical School, Boston, Massachusetts, United States of America; University of Missouri-Columbia, United States of America

## Abstract

Keratoconus (KC) is a bilateral degenerative disease of the cornea characterized by corneal bulging, stromal thinning, and scarring. The etiology of the disease is unknown. In this study, we identified a new biomarker for KC that is present *in vivo* and *in vitro*. *In vivo*, tear samples were collected from age-matched controls with no eye disease (n = 36) and KC diagnosed subjects (n = 17). Samples were processed for proteomics using LC-MS/MS. *In vitro*, cells were isolated from controls (Human Corneal Fibroblasts-HCF) and KC subjects (Human Keratoconus Cells-HKC) and stimulated with a Vitamin C (VitC) derivative for 4 weeks, and with one of the three transforming growth factor-beta (TGF-β) isoforms. Samples were analyzed using real-time PCR and Western Blots. By using proteomics analysis, the Gross cystic disease fluid protein-15 (GCDFP-15) or prolactin-inducible protein (PIP) was found to be the best independent biomarker able to discriminate between KC and controls. The intensity of GCDFP-15/PIP was significantly higher in healthy subjects compared to KC-diagnosed. Similar findings were seen *in vitro*, using a 3D culture model. All three TGF-β isoforms significantly down-regulated the expression of GCDFP-15/PIP. Zinc-alpha-2-glycoprotein (AZGP1), a protein that binds to PIP, was identified by proteomics and cell culture to be highly regulated. In this study by different complementary techniques we confirmed the potential role of GCDFP-15/PIP as a novel biomarker for KC disease. It is likely that exploring the GCDFP-15/PIP-AZGP1 interactions will help better understand the mechanism of KC disease.

## Introduction

Keratoconus (KC) is an ectatic eye disease associated with structural abnormalities in the cornea [1]. KC is characterized by a central or paracentral stroma thinning, corneal bulging, and scarring [1]–[3]. Other characteristics include the appearance of Fleischer’s ring, interruptions in Bowman’s layer, decreased keratocyte density, and severe changes in collagen gross organization [4]. KC typically appears in teens and progresses until the third or fourth decade of life [5]. The severity of the disease and how this is reached varies considerably between individuals. Ultimately, vision quality is compromised and corneal transplantation is required in severe cases.

The pathophysiology of KC is still unknown and early asymptomatic onset of the disease progresses freely [1], [6], [7]. Once KC has reached a certain level diagnosis can be made by corneal imaging techniques either by documentation of an irregular increase in the refractive power of the anterior cornea or a localized thinning of the corneal stroma, but most often a combination of these finding [7], [8]. Individuals diagnosed with early KC are regularly prescribed with contact lenses, not for treatment but to maintain good vision [7]. The progression of keratoconus can in most of the milder cases be halted by corneal cross-linking [9]–[11], which is used in most countries, although FDA-approval is still ongoing in the US. Advanced stages require corneal transplantation.

Despite significant efforts, a sensitive and specific biomarker for keratoconus has not been identified. Early identification of subjects prone to develop keratoconus would be useful in identifying subjects, which should be followed with detailed corneal imaging, as corneal cross-linking recently has been documented to halt disease progression in keratoconus. A marker would also be very useful in screening patients seeking corneal refractive surgery. After laser surgical procedures, a small number of patients are known to develop “iatrogenic ectasia” which is very similar to naturally occurring keratoconus. In this study we propose a potential new biomarker for the identification of KC. Gross cystic disease fluid protein-15(GCDFP-15) also known as prolactin-inducible protein (PIP) is a secretory glycoprotein of 14 Kda [12] which others have found expression of in the proteome of human tear fluids [13]. Quality of tear fluids are key in maintenance of healthy cornea structure and the vast majority on KC studies are concentrating on analyzing the tear proteome between individual cases.

We have recently reported a 3D culture system for studying human keratoconus cells (HKC) [7], [14]. We have reported findings that are in agreement with what is seen in vivo, such as increased oxidative stress levels in HKCs when compared to normal human corneal fibroblasts (HCF) [11]. Here we collected human tear samples and using proteomics analysis we identified PIP as a potential biomarker for KC disease. In order to validate our findings and integrate our in vivo data with the in vitro model we used our 3D in vitro culture model. The regulation of PIP was validated using real time PCR and western blot analysis. To the authors knowledge this is one of the first studies to identify a potential biomarker for KC disease that is regulated similarly both in vivo and in vitro.

To reinforce our findings, Zinc-alpha-2-glycoprotein (AZGP1), a gene that is known to stimulate breakdown of lipids (lipolysis) [15], [16] and bind to PIP was also found to be significantly regulated both in vivo and in vitro. This indicates that the interplay between the two proteins (PIP-AZGP1) might provide clues to KC pathogenesis.

Further understanding of the mechanism of how PIP is regulated is clearly needed. We present evidences that one of the main growth factors involved in corneal wound healing as well as KC disease; transforming growth factor-β (TGF-β), can significantly regulate PIP and its expression. This could potentially have major implications in the prevention and prognosis of KC disease.

## Methods

### Subject recruitment

36 patients referred to the Department of Ophthalmology, Aarhus University Hospital for keratoconus was asked to provide a tear sample for testing. All patients underwent a standard clinical examination including refraction, measurement of best corrected visual acuity, slit-lamp examination, and Pentacam HR Scheimpflug tomography. 17 patients referred for refractive surgery for myopia underwent similar examination and served as control group. Tears were collected in capillary glass tubes from the lateral tear meniscus. Care was taken not to stimulate tear secretion during collection.

### Ethics

Tear collection was considered part of the highly specialized clinical and para-clinical evaluation of patients referred to the department. Initially, tear samples were collected for methods development. The Regional Ethics Committee for the Central Region of Denmark approved this study and written consent was obtained from the study participants. The approval reference number is: 1-10-72-77-14. The study met the tenets of the Declaration of Helsinki. Tear samples were anonymized before analysis.

### Proteomics-tandem mass spectrometry

As previously reported by us and others, all tear samples were analyzed by microcapillary liquid chromatography-tandem mass spectrometry (LC-MS/MS) [11], [17], [18] using the EASY-nLC nanoflow HPLC (Thermo Fisher Scientific; Waltham, MA) with a 75 µm inner diameter × 15 cm length C_18_ capillary column coupled to a hybrid LTQ Orbitrap XL-ETD mass spectrometer (Thermo Fisher Scientific). Label-free quantification was used to quantify and determine the differential expression levels of proteins between samples. The high-resolution power facilitated the extraction of peptide signals on the MS level, thus uncoupling the quantification from the identification process [11], [17], [18]. Proteins that were not expressed in the majority of the samples, were excluded from our quantification.

### Isolation and primary cells

HCFs were isolated from human corneas from healthy patients without ocular disease. All samples were obtained from NDRI (National Disease Research Interchange; Philadelphia, PA; http://www.ndri.org/) as previously described [11], [14], [19]. HKCs were isolated from human corneas from anonymized patients undergoing corneal transplantation for keratoconus at Aarhus University Hospital, Aarhus, Denmark. The research adhered to the tenets of the Declaration of Helsinki. The tissue was obtained and used for scientific purpose and ethical issues have been handled according to Danish healthcare law (research in anonymized biological material which is normally discarded), and after guidance from the local ethical committee (The Regional Ethics Committee for the Central Region of Denmark), and in accordance with the Declaration of Helsinki.

As previously described [19] the corneal epithelium and endothelium were removed from the stroma by scraping with a razor blade. The stromal tissues were cut into small pieces of size ∼ 2×2 mm and placed into T25 culture flaks. Explants were allowed to adhere to the bottom of the flask at 37°C for about 30 minutes and Eagle’s Minimum Essential Medium (EMEM: ATCC: Manassas, VA) containing 10% fetal bovine serum (FBS: Atlantic Biological’s; Lawrenceville, CA) and 1% Antibiotic (Gibco Antibiotic-Antimycotic, Life technologies) was added carefully without disturbing the implants. Further, the cells were passaged into T75 culture flasks upon 100% confluence after 1–2 weeks of cultivation at 37°C, 5%CO_2._

### Culture and assembly of ECM

Both HCFs and HKCs cells were cultured on 6-well tissue culture plates and processed for q RT- PCR and western blot analysis [19], [20]. 1×10^6^ cells/well of these cells was seeded and cultured in EMEM 10%FBS medium stimulated with 0.5 mM 2-O-α-D-Glucopyranosyl-L-Ascorbic Acid (Vit C, American Custom Chemicals Corporation, San Diego, CA). Cells were further stimulated with one of the three TGF-b isoforms: TGF-β1 (T1), TGF-β2 (T2) and TGF-β3 (T3). All isoforms were used at 0.1 ng/mL concentration as previously optimized [19]–[21]. The cultures were grown for four weeks before further processing. Cultures without any growth factors served as the controls (C). Fresh media was supplied to the cultures every other day for the whole duration of the experiment.

### Real Time PCR

For evaluation of the m RNA expression, q RT-PCR was done on all samples as previously described [19], [21]–[23]. Briefly, total RNA extraction was carried out using Ambion RNA mini extraction kit (Ambion TRIzol Plus RNA Purification Kit**:** Life technologies, Carlsbad, CA). The cDNA synthesis was followed using a SuperScript III First-Strand Synthesis SuperMix (Invitrogen, Carlsbad, CA) according to the manufacturer’s protocol. TaqMan gene expression assay (Applied Biosystems, Foster City) GAPDH (Hs99999905_m1) and 18S (Hs99999901_s1) was used as control and PIP (Hs00160082_m1) and AZGPI (Hs00426651_m1) as study probe. The reaction was set up using 10 ng of cDNA in a 20-µl reaction containing our probe of interest and Taqman FaSt Advanced Master Mix (Applied Biosystems, Life technologies, Foster city, CA). StepOnePlus real-time PCR system (Life Technologies, Foster city, CA) was used for amplification of the sample using standard manufacturer’s protocol. Graph Pad Prism 6 and MS-Excel was used for data analysis. All samples and probes were repeated at least three times.

### Western blot

Cell lysates for HCFs and HKCs were prepared for western blot analysis, as previously described [21], [24]. Briefly, bradford assay (Thermo scientific, IL, USA) [21] was carried for determining the protein concentration and purity. Samples were mixed with loading buffer and equal amounts of protein were loaded on readymade 4–20% Tris-Glycine gel (Novex, Life technologies, Carlsbad, CA) for gel electrophoresis. Proteins were transferred on to a nitrocellulose membrane (Novex, Nitrocellulose membrane filter par sandwich, Life Technologies, Carlsbad, CA). Thereafter the membranes were incubated in two different blocking solutions (5% milk in TBST; Thermo scientific, IL, USA) and (5% BSA in TBST) as per the manufacturer’s antibody specification protocol; for 1 hour and incubated overnight at 4°C with primary antibody (Rabbit Monoclonal Anti-GCDFP 15 and Rabbit polyclonal Anti-Zinc Alpha 2 Glycoprotein, Abcam, Cambridge, MA) at 1∶1000 dilutions. Thereafter membranes were washed and incubated with a secondary antibody (Alexa Flour 568 Donkey Anti-Rabbit, IgG (H+L), Abcam) with 1∶2000 dilutions for 1 hour. The binding of antibodies to the membrane was detected with Kodak imaging system. Result were analyzed by normalizing the value to that of the house keeping antibody GAPDH (Abcam, Cambridge, MA) expression and the fold expression was plotted.

### Statistical Analysis

Data analysis for the sample sets, n = 3 was done by one -way ANOVA using Graph Pad Prism 6 software. Where P value (P<0.05) was considered to be statistically significant.

## Results

### Pentacam

As shown in Table 1 the mean age for healthy individuals was 33 (range from 18 to 47 years). For Keratoconus individuals the average age was 30 (range 21 to 59 years). There were no statistically significant differences in age between the study groups. All individuals participating in the study were examined using the Pentacam. A variety of values were collected. The mean corneal thickness for the control group was 501.2, and for the keratoconus group was recorded as 466 (Table 1). Maximum keratometric (Kmax) average value for the controls was 46.9, and the keratoconus group average value was 53.9 (Table 1).

**Table 1:** Pentacam data collected from participating subjects.

	Age (years)	Ct min (um)	Kmax (D)
**Control**	33±8.58	501.2±8.94	46.9±4.69
**Keratoconus**	30±10.58	466±4.77	53.9±7.79

Summary of the mean ages, corneal thickness (Ct min) and maximum keratometric (Kmax) values for: Controls and Keratoconus. Mean age for Control was 33 and for KC the average age was 30. No statistically significant differences were found. The Ct min for Controls was 501.2 and KC was recorded as 466. There was a statistically difference between the two groups (p<0.05). Kmax average value for the controls was 46.9 and the KC average value was 53.9.

### Proteomics analysis

All tear samples were analyzed by microcapillary liquid chromatography-tandem mass spectrometry (LC-MS/MS). Our study confirmed the findings of previous studies with some of the most common proteins regulated in KC tear samples reported in table 2. Lipophilin-A, Immunoglobulin J Chain, Cystatin-S, and Lactotransferrin were all significantly down regulated (p<0.0001) in KC tear samples. AZGP1, a closely related protein to PIP was slightly up regulated in KC but did not reach significance (p = 0.18).

**Table 2:** Selected proteins regulated in tears. * indicates statistical significance.

	Normal	Keratoconus	P value
Lipophilin-A	3.44E+08±6.15E+07	2.48E+04±1.82E+03	P<0.0001*
Immunoglobulin J Chain	4.85E+08±1.69E+08	2.10E+04±1.83E+03	P<0.0001*
Zinc-alpha-2-glycoprotein (AZGP1)	2.86E+08±1.06E+08	5.82E+08±1.76E+08	P = 0.18
Cystatin-S	6.50E+08±2.30E+08	1.90E+04±1.13E+03	P<0.0001*
Prolactin-inducible protein (PIP)	1.45E+09±4.83E+08	2.63E+04±3.34E+03	P<0.0001*
Lactotransferrin	1.40E+10±4.30E+09	2.12E+04±9.43E+02	P<0.0001*
Secreted frizzled-related protein 1	1.28E+06±5.26E+05	2.87E+06±5.27E+05	P = 0.076

Summary of the most common proteins regulated in KC tear samples. Lipophilin-A, Immunoglobulin J Chain, Cystatin-S, and Lactotransferrin were all significantly down regulated (p<0.0001) in KC tear samples. No significant changes in the levels of Secreted frizzled-related protein 1 and AZGPI (p<0.076 and p = 0.18 respectively) was found. PIP was significantly down regulated (p<0.0001) in KCs.

### mRNA expression

**Figure 1:**
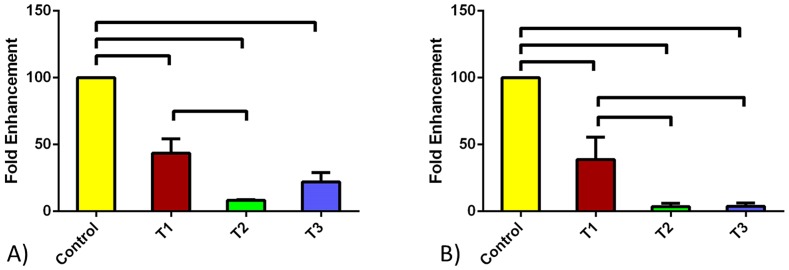
PIP quantified mRNA expression for A) HCF and B) HKC cell types. Four conditions were tested: Control, TGF-β1, TGF-β2, and TGF-β3. All three TGF-β isoforms resulted in significant down regulation of PIP for both cell types. All error bars represent standard error (SEM) of n = 3.

We quantified the mRNA expression levels of both PIP and AZGPI in the HCFs and HKCs. Figure 1 shows the PIP expression under four different conditions; Control, TGF-β1, TGF-β2, and TGF-β3. Upon treatment with the three TGF-β isoforms, levels of PIP were significantly lower (p<0.0001) for both cell types (Figure 1A and 1B). TGF-β2 and TGF-β3 almost shut down expression of PIP in HKCs.

**Figure 2:**
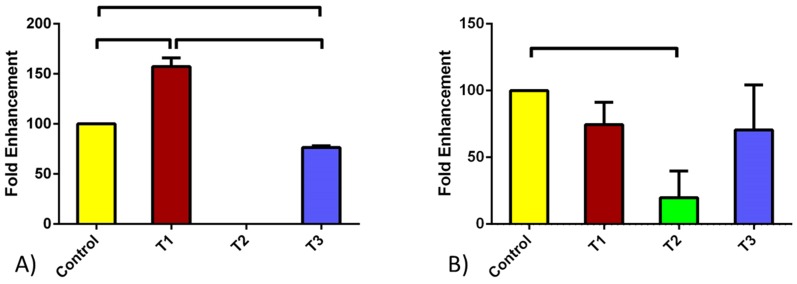
AZGPI quantified mRNA expression for A) HCF and B) HKC cell types. Four conditions were tested: Control, TGF-β1, TGF-β2, and TGF-β3. TGF-β1 led to an increase of AZGPI in HCFs where no effect was seen in HKCs. When cells were stimulated with TGF-β2 no expression was found in HCFs, where HKCs expression was significantly down regulated when compared to the Controls. Upon TGF-β3 treatment AZGPI expression was significantly down regulated for HCFs while it remained unchanged for HKCs. All error bars represent standard error (SEM) of n = 3.

The mRNA expression of AZGPI was also tested due to the interplay between the two genes. Figure 2 shows expression of AZGPI in HCFs (Figure 2A) and HKCs (Figure 2B). TGF-β1 led to significant up regulation of AZGPI in HCFs (Figure 2A; p<0.0005) while no change was noted in HKCs. This was opposite to the PIP regulation with TGF-β1 (Figure 1). TGF-β3 on the other hand down regulated AZGPI expression in HCFs. Again no effect was noted in HKCs. Interestingly, when cells were stimulated with TGF-β2 no expression was found in HCFs, where HKCs expression was significantly down regulated when compared to Controls (Figure 2B; p<0.05).

### Protein expression

**Figure 3:**
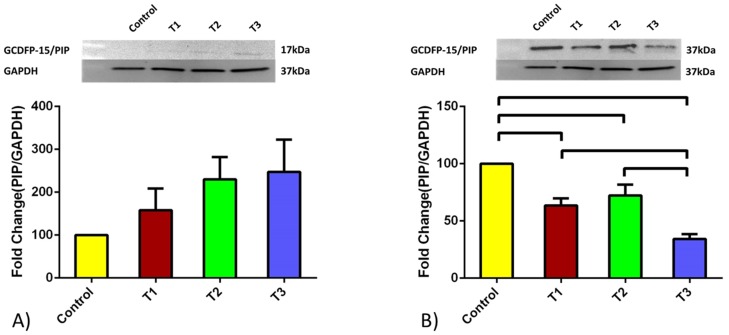
PIP quantified protein expression for A) HCF and B) HKC cell types. Four conditions were tested: Control, TGF-β1, TGF-β2, and TGF-β3. HCFs expression was increased upon TGFβ treatments however none of them reached significance. HKCs showed significant down regulation upon all TGFβ treatments. TGFβ3 showed the lowest PIP protein levels compared to all other conditions. HKCs PIP protein levels were consistently lower under all conditions when compared to HCFs. Note: HCF’s PIP expression was seen at 17 kDa protein whereas in HKC’s at 37 kDa. All error bars represent standard error (SEM) of n = 3.

**Figure 4:**
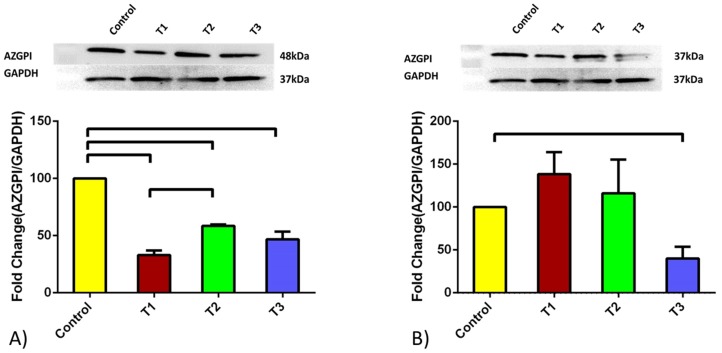
AZGPI quantified mRNA expression for A) HCF and B) HKC cell types. Four conditions were tested: Control, TGF-β1, TGF-β2, and TGF-β3. In HCFs all three TGFβ isoforms resulted in significant down regulation of AZGPI expression. TGFβ1 showed the lowest of the three when compared to the controls. In HKCs, no significance changes in AZGPI expression was shown for TGFβ1 and TGFβ2 isoforms. TGFβ3 stimulation significantly down regulated AZGPI protein expression. All error bars represent standard error (SEM) of n = 3.

We examined the protein expression of both PIP and AZGPI for both cell types. Figures 3 and 4 shows the fold enhancement quantified data obtained for HCFs and HKCs respectively. Values were normalized to the expression of the controls of the respective cell type. Opposite to the mRNA levels, HCFs PIP protein expression was up regulated upon all TGFβ treatment (Figure 3A) however none of them reached significant values (p = 0.2457). However, HKCs showed identical regulation at the protein level (Figure 3B) as with the mRNA level (Figure 1B), where PIP was significantly down regulated upon TGFβ stimulation. All isoforms resulted in PIP protein down regulation compared to Controls (TGFβ-1: p<0.05, TGFβ2: p<0.05, and TGFβ3: p<0.001). As with the mRNA expression, PIP protein levels were lower with TGFβ3 treatment (Figure 3B). Of note here, HCF’s PIP protein expression was seen at 17 kDa protein whereas in HKC’s at 37 kDa. This higher kDa protein expression indicates the presence of a glycosylated version of the protein.

The expression pattern for AZGPI in HCFs was rather similar to that of HKCs (Figure 4A). In HCFs all three TGFβ isoforms resulted in significant down regulation (p<0.0001) of AZGPI protein expression with TGFβ1 showing the lowest of the three when compared to the controls (Figure 4A). This is in agreement with our mRNA levels regulation. In the case of HKCs, treatment with TGFβ1 and TGFβ2 isoforms showed identical AZGPI protein expression, whereas treatment with TGFβ3 greatly resulted in decreased protein expression (Figure 4B; p<0.05). Overall the protein expression and mRNA levels seem to be similarly regulated upon TGFβ stimulation; especially with TGFβ3. These results confirm the prognostic properties of TGFβ3 during diseased condition. Similar to what was seen with PIP, HCF’s expression of AZGP1 was seen at 26 kDa where HKCs at 37 kDa.

## Discussion

Keratoconus is a progressive degenerative disease and a major clinical problem worldwide [11]. The disease can progress rapidly starting at puberty [19] and can lead to blindness if not treated. Early clinical detection of this disease is a challenge and usually signs and symptoms are similar to those seen in severe astigmatism. One of the major limitations for the understanding of the disease is the unavailability of an animal model as well as an *in vitro* model. In 2012 [19] we proposed a novel 3D culture system for studying HKC in vitro. We have characterized and continue to develop this model in order to dissect the mechanisms of KC. Clearly, substantial work still remains in order to identify the exact etiology for the onset of disease.

KC studies have suggested that genetic, environmental, and molecular factors might be playing an important role. All of these studies have indicated that specific biomarkers for early detection of the disease, which would allow early treatment, are missing. Over the last decade, studies have concentrated on tear fluids for identification of such a biomarker. The rationale behind thes studies have been that the quality of tear fluids are considered to be the key in maintenance of healthy cornea structure [25]. Some studies have suggested that the imbalance in presence of cytokines, enzymes, enzyme inhibitors and other mediators in the tear fluid might be playing an important role in the development of the disease [26]. Others have shown evidence of oxidative stress [11] with altered antioxidant enzymes [27]–[30], accumulated byproducts from lipid peroxidation, nitric oxide pathways [31], [32] and damaged mitochondrial membrane potential [33], [34]. Biomechanics and collagen structure has also been investigated by scientists. A variety of techniques have been used in order to compare normal and keratoconus corneal stroma structure. Multiple groups have used X-ray scattering and scanning electron microscopy [35]–[38] to map the collagen orientation and fibrillar mass in keratoconus corneal buttons. Meek and co-authors, in a more advanced approach, reported the relationship between corneal shape and thickness in keratoconus samples using combination of videokeratography and X-ray scattering by Hayes et al [39].

In this study, using both in vivo and in vitro systems, we report GCDFP-15/PIP as a potential novel biomarker possessing predictive and prognostic effect for KC. GCDFP-15/PIP is a secretory acinar protein of 14–17 kDa molecular weight, which is normally secreted in various body fluids [12], [40]–[43], and has already been demonstrated to be a novel biomarker in cancer and several other diseases [13], [22]–[26]. These studies have reported expression of this protein at various levels with variable levels of glycosylation.

In our in vivo proteomics analysis we found proteins whose levels are altered in KC compared to healthy individuals with no history of ocular disease. These included Lipophilin A, Immunoglobulin J chain, Cystatin S, Lactotransferin and secreted frizzled-related protein 1. Our proteomics data also showed regulation of PIP. PIP is closely related to AZGPI protein, which in our group of patients was not significantly altered. However, the binding relationship PIP at a similar region encouraged us to consider it further. It has been reported that AZGPI and PIP forms a complex [15] in some body fluids and they localize in close vicinity in the same chromosome [44]–[46] and are both regulated by androgens [15]. AZGPI which is also a glycoprotein has been found to stimulate lipolysis [28] because of its ability to play many important function in the human body it has been considered as a potential bio marker for various studies especially for cancer related research [22], [28]. AZGPI has been reported to be a protein of 35–44 kDa.

We confirmed the importance of PIP and AZGPI using our 3D in vitro model. Our previous studies on HCKs and HCFs using this model have been concentrated on the three TGF-β isoforms and their significance in corneal fibrosis effects and elevated ECM production [19]–[22], [47]–[49]. We have shown that TGF β3 is anti-fibrotic in HCFs and promotes ECM assembly similar to that seen in vivo. We therefore investigated their role on regulating PIP and AZGPI in vitro, and determined a higher m RNA for GCDFP-15/PIP in HCFs in comparison to HKCs. Upon TGF-β3 treatment, the expression pattern decreased in comparison to the HCFs. The m RNA expression for the AZGPI genes showed a similar pattern of expression to that of the PIP for TGF-β isoform treated samples. Interestingly, no m RNA expression was seen with TGF-β2 treatment in HCFs when compared to HKCs. This suggests the possible masking or inhibition effect of the growth factor on the gene expression. In order to further investigate this and evaluate the PIP as a biomarker we performed a western blot analysis on all our samples.

Both HCFs and HKCs showed a sequentially increased level of protein expression with the treatment of TGF-β isoforms when compared to the controls. Interestingly, HCF’s GCDFP-15/PIP expressed a 17 kDa protein whereas in HKC’s a 37 kDa protein expression was observed. The higher kDa protein expression suggests the presence of a glycosylated version of the protein [12], [15], [50]. Clinically this would suggest that several mediators secreted in KC tears like cytokines and enzymatic inhibitors [26] bind to the PIP protein and result in significant drift to a higher molecular weight. The exact biological function of PIP is not clear, however, it has been reported to bind many proteins like fibrinogen, actin, keratin myosin, and CD34 molecule of T cells [12], [40]. Predicting the reason for the higher molecular weight protein expression at this stage is rather challenging and future analysis of this protein will be needed to provide a better picture of the exact specificity and nature of the protein.

Overall, it is evident from our data that both genes were significantly decreased by TGF-b3 treatment in HKCs compared to the HCFs, This indicates PIP/AZGPI can certainly be a prognostic indicator during the pathogenesis of the disease. AZGPI has been reported to regulate lipid metabolism, induce lipolysis, and down regulation in obese patients [51]. This provides strong evidence that it is strongly associated with body metabolism and secretion of different amounts of metabolites might be directly or indirectly engaged in deciding the expression pattern of the genes. This would agree with our previous findings and the role of TGF-β3 in HKCs [11].

GCDFP-15/PIP and AZGPI both are present in the same chromosome and both inaccessible from the N-terminal region due to the presence of pyroglutamine [15]. This gives them almost identical structure and nature. Furthermore, they both bound to the nucleus and the membrane region and have been identified to possess a remarkable binding pattern to each other [43]. In addition, both are involved in cell cycle regulation, having effect on proliferation and inhibition of cells and they are also considered to be present in the same overlapping signaling pathways [52].

Further studies are clearly needed in order to understand their role in KC as well as their relationship to TGF-β isoforms. Previous studies have reported activation of PIP and AZGPI via cAMP signaling by ERK-dependent pathway. Therefore, we predict that due to the same binding zones and strong affinity for each other, the possible activation pathway for both the genes could be STAT5. STAT which is a latent cytoplasmic protein activated upon receptor stimulation is found to be mediated by protein tyrosine kinases. It has been reported after phosphorylation with tyrosine the STAT pathway acquires binding ability and translocate to the nucleus by binding to the specific promoter elements and controls the expression of the target genes. Their involvement in various pathological conditions has been well established [53]–[55].

In conclusion, it can be strongly stated that both GCDFP-15/PIP and AZGPI hold great promise as novel prognostic and predictive biomarkers for KC and can provide various clues at various levels of disease progression. This is the initial report of these two genes as biomarkers for KC. Further extensive research of the possible pathways and gene expression patterns of these markers might lead to determining the pathological treatment for KC.
